# Collectanea: Pathology and Practice

**Published:** 1834-01-01

**Authors:** 


### COLLECTANEA.

### PATHOLOGY AND PRACTICE.

### REMARKABLE CASE OF ASCITES.

A JOINER'S wife, about thirty years old, had been subject since her childhood to a remarkable hardness of the abdomen, without suffering any other inconvenience. This state had lasted for about fifteen years, and she had used a variety of remedies with the intention of bringing down her belly; she had been long married, but had never borne a child. I was accidentally consulted by her, as I was attending her husband, who was labouring under pleurisy. The catamenia were regular, but she suffered from fluor albus vaginæ; the abdomen was of almost stony hardness. The evacuations by stool and urine were normal, her appetite good, and her complexion healthy; there were no symptoms of any affection of the liver or spleen, no trace was present of scrofulous cachexy. I thought that the case depended on a swelling between the parietes of the abdomen and the peritoneum, or perhaps on a steatomatous or fungous tumour. Several physicians, to whom I communicated the case, agreed in my diagnosis. What was to be done? I gave pills of Extr. Taraxaci, Graminis, Sulph. Aurat. Antimonii, and Aloes, to see if collections of mucus were present, as they often are, according to my experience, in persons who are apparently quite healthy, as they do not always betray their presence by such especial symptoms as are given in the books. Accordingly, great masses of mucus were passed by stool for three weeks, without any decrease in the abdominal swelling, or any other alteration; I then desisted from the employment of these remedies, and told the patient that her cure, if it took place, could be effected only by a long course of treatment. She would not consent to this, as her disease caused her no inconvenience. For two years I did not hear from the patient, though I often saw her walking about, when I suddenly heard that she was ill in bed, and that a surgeon had said, she had water in her belly, and must be tapped, for which purpose a physician was to be fetched from Rostock. The thing however was not done, and after the patient was going about again, I learned from the surgeon just mentioned, that an opening had formed itself in the abdominal parietes, out of which water flowed, and that there was an obvious fluctuation around this opening, though I could not discover any, when I attended her; the surgeon therefore advised that the opening should be enlarged, and a trocar inserted, and I perfectly agreed with him. The operation was put off, but was performed about six months afterwards by a physician of this place, (Rybnik,) who was called into consultation, and a great quantity of water was evacuated. The abdominal cavity contracted, and the opening closed; and two years have now elapsed, since which the patient is quite well, and the abdomen is of a natural softness. It may be asked, how was it possible that an ascites should last so many years, without bad consequences? My opinion is, that it was a hydrops saccatus, and that the sac at last burst, as well as the parietes of the abdomen. So that it was a genuine cure performed by nature. Have other physicians and surgeons seen any thing similar?—*Dr. Tott, in Gräfe and Walther's Journal*, Band xix. Heft 3.

[Yes; this was a case of ovarian dropsy, in which the water was effused into the cavity of the abdomen; and Dr. Tott will find two similar cases related by Dr. H. Davies, in our last number.]—
EDIT.

### ON THE USE OF THE SULPHATE OF CADMIUM IN DISEASES OF THE EYE.

A minute fragment of iron, about half as big as a pin's head, sprang into the left eye of a country boy, aged ten, as he was standing in a smithy. Dr. Tott, who narrates the case, did not see the patient till a fortnight afterwards, when he was suffering from inflammation of the conjunctiva and cornea, no remedies having been used in the interval. Dr. Tott ordered the eye to be shaded from the light, leeches to be applied, and aqua saturnina to be dropped in. No trace could be discovered of the fragment of iron. In eight days the inflammation and the swelling of the conjunctiva had somewhat diminished, and the piece of iron could be plainly seen, in a small fold, formed by the swollen conjunction of the cornea. It was removed with a pair of eye-forceps, leeches, and saturnine lotion were repeated, and a purgative was given, consisting of calomel and jalap. The inflammation soon disappeared; but the boy's sight was clouded by a blueish spot on the cornea, which covered half the pupil. Mercurial ointment, a collyrium of sulphate of zinc, walnut oil, and a perpetual blister behind the ear of the diseased side were employed without advantage; but eight or ten drops of a collyrium of the sulphate of cadmium, (made with gr. j. to ℥ij. of distilled water,) being now introduced into the eye three times a day, the speck was removed in four weeks, and the boy's sight perfectly restored.

The second case occurred in the same village in Pomerania as the former one. The patient was a girl about five or six years old, who had poked something into her right eye, thus causing a traumatic inflammation of the conjunctiva and cornea. Dr. Tott, who was not called in till six weeks after the accident, found that partial exudation had taken place, and that a speck had formed upon the cornea. Instructed by the event of the case just detailed, Dr. Tott used the sulphate of cadmium in the same manner, and in six weeks no trace of a speck was to be discovered in the eye of the little patient.

Both cases show that specks on the cornea caused by external violence can be removed by the same remedies which cure those which have arisen from internal causes. Hence, it is erroneous to suppose that the former cases are more difficult to cure than the latter, in which it was thought that the external remedies could be assisted by medicines intended to remove the internal cause. But the following instance shows, that even in the latter class of cases, cadmium, by itself, can effect a cure :

A boy, aged five, was suffering from a bark-like eruption on his face, of scrofulous origin, and at the same time from inflammation of the left eye. Dr. Tott administered antimonials, Plummer's powder, dulcamara, and conium, and the eruption disappeared; the ophthalmia also yielded to appropriate remedies, but a blueish-white spot remained on the cornea and covered the pupil. Blisters placed behind the ear, and collyria of zinc and lead, were of as little benefit as the continued use of antimonials and mercurials, from which Dr. Tott says he promised himself great advantage, on account of the symptoms of a scrofulous dyscrasia, which still remained. Afterwards tartar emetic ointment was rubbed in behind the ear; but this as well as all the other remedies having been discontinued, the sulphate of cadmium was now employed, and in a week had already lessened the speck. In six weeks more it would have disappeared entirely, but the parents withdrew the patient from my care, says Dr. Tott, for fear of greater expense; but it is probable that the speck, which was very small, and scarcely any hindrance to vision, is now entirely removed. It is not probable that the antiscrofulous remedies contributed to the cure, since even the eruption appeared again, and all the other symptoms of a scrofulous dyscrasia continued. When inflammations depend on an internal dyscrasia, then internal remedies are necessary; but when inflammation has run through its cycle, and terminated in exudation, the speck is then a purely local relic of the cachectic inflammation; it is not itself of a cachectic nature, but is to be looked upon as a disease no longer depending on an internal cacochymia, and therefore to be treated merely by local remedies. The preceding cases show the sulphate of cadmium to be an excellent topical remedy, though unfortunately it is seldom used, even by those physicians who are especially devoted to the treatment of ophthalmic diseases; they talk continually of the employment of zinc, vitriolic remedies, corrosive sublimate, red precipitate, &c.; and when these means have failed, as often happens, they complain that nothing was of any use, and that the disease is a *scandalon artis*. Dr. Tott, however, confesses, that in a former number of the journal he mentioned a case of leucoma corneæ in which the red precipitate ointment bore off the palm from the sulphate of cadmium: but he is convinced that the remedy as applied by him then was not so strong as what he now employs.—*Gräfe und Walther's Journal*, Band xx. Heft 2.

### SUDDEN COMPLETE AMAUROSIS, FROM VIOLENT INFLAMMATORY ATTACK OF THE BRAIN AND MEMBRANES.

A patient, thirty-seven years of age, came under my care at St. Bartholomew's Hospital, on the 2d of March, 1832, on account of inflammation of the basilic vein, consequent on venesection: the affection had begun on the 29th of January. The symptoms, which were serious and alarming, subsided under active treatment; the phlebitis had completely disappeared by the 16th of March, when the circulation was tranquil, the rest sound, the tongue clean, and the appetite good.

A mutton-chop was ordered daily, at the urgent request of the patient, who felt very hungry; and a draught of infusion of cascarilla, with infusion of rhubarb, three times a day. He took the chop, and three draughts, on the 17th, slept well, and had the bowels opened on the 18th, when he rose and dressed himself, and was found by the dresser sitting by the fire. He said he felt so well, that he could not remain in bed; he complained of being very hungry, and requested that he might have wine or beer at dinner: this was immediately refused. In the course of the morning his friends came to see him, and it was strongly suspected that they had brought him wine or spirits. He could not eat the chop at dinner; but he made no complaint at that time. At six P.M. he was suddenly seized with faintness, loss of sight, and slight pain in the head. He went to bed immediately, and had some calomel and James's powder, followed by an ounce of castor-oil in four hours.

19th. Restless and delirious during the whole night; the retinæ are totally insensible, so that he cannot distinguish light from darkness. The pupils are slightly dilated, but contract when a candle is held before the eyes, although he is not aware of its presence. The head is hot and painful. He is sensible, and answers questions rationally. The pulse small and quick; tongue white, and rather dry; there is great thirst.—The head to be shaved, and covered with cold lotion; cupping on the nape to fourteen ounces; a blister between the shoulders; a saline draught, with antimonial wine, every four hours.

20th. Vision the same.—Twenty-four leeches to the temples; a dose of castor-oil; two grains of calomel, with two of antimonial powder, every four hours.

21st. A little sleep towards the morning; vision improved, so that he can see fingers held before him, and tell the number. In the middle of the day the former symptoms returned in an aggravated degree; delirium came on, with continual muttering, and the stools were passed unconsciously.—Blisters to the calves.

Death took place on the 23d. Seropurulent effusion was found in the pericardium, and purulent infiltration to a considerable extent had taken place in the muscular substance of the heart. Unequivocal evidences of vascular excitement were found throughout the encephalon; the medullary substance was partially softened in several situations. The arachnoid membrane was thickened at the basis of the brain, and yellow coloured, apparently from purulent infiltration. These changes were particularly conspicuous about the infundibulum, and the union of the optic nerves.—*Lawrence on Diseases of the Eye.*

### COMPLETE AMAUROSIS, WITH CONGESTION IN THE HEAD, IN A PLETHORIC SUBJECT: PERFECT RECOVERY.

A young woman, twenty years of age, came to the London Ophthalmic Infirmary, then in Charter-house square, labouring under active congestion about the head; her countenance was peculiarly florid; all the veins were obviously turgid, and she had considerable pain. Before I saw her she had experienced severe external inflammation of one eye, which had produced a large leucomatous opacity of the cornea: the latter was in the state of incipient staphyloma. These serious changes, and the consequent loss of an eye in a young female of considerable beauty, were the obvious results of inactive treatment. She still suffered occasional relapses of inflammation in the eye; and at these times she experienced so much sympathetic affection of the opposite eye, that she could not use it without pain. Hence she readily submitted to the removal of the staphylomatous projection, which quickly and effectually relieved the sound eye. Within a month she came again, saying that she had lost the sight of the other eye. On examining it, I found that she had an attack of almost complete amaurosis: although it had existed only two or three days, she could scarcely distinguish the window. She had a dilated pupil and nearly motionless iris; there was no external redness, but she had, in addition to her usual florid colour, a flushed countenance, considerable pain in the head, and some febrile disturbance of the system. Knowing from experience how prone she was to inflammatory attacks in the eye, I had her bled largely, and cupped from the back of the neck, placed her upon low diet, and gave purgative medicines; but, although this plan of treatment was followed up vigorously, no improvement of vision ensued; the retina indeed became quite insensible. Mercury was now used actively: as soon as it caused salivation the affection began to give way, and she recovered her sight perfectly. She was still liable to returns of congestion about the head, and repeatedly lost blood by venesection and cupping: she continued to employ mercury and aperients, and ultimately had a seton in the back of the neck. In spite of such means, continued and repeated for more than a year, during which time she was often bled and cupped, and took an immense quantity of mercury and purgatives, she still retained her beautiful colour and the florid red of a person from the country. She attended the Infirmary nearly a year and a half, undergoing the treatment above described, more or less actively, during the whole time. I saw her two years afterwards, the eye remaining perfectly well; but, although she had still much colour, the characters of youth and beauty were in a great measure lost.*—*Ibid.*

### POST-MORTEM CONVULSIONS IN CHOLERA PATIENTS.

An artillery man, named Kapienski, aged twenty-three, a tall man, of herculean strength, and tolerable *embonpoint*, died about noon on a warm September day, after an eight hours' illness. I observed his corpse, (the convulsive movements of which had already been remarked by the attendant,) from half past one to three in the afternoon. Single muscles in the upper-arm, forearm, and lower part of the thigh, moved convulsively, at intervals of one, two, or more minutes, so, as occasionally to move a finger, and more rarely a toe; the motions of the lower extremities being rarer and weaker. The body had the usual posture, which I have already described; the fingers being moderately bent, and the ordinary motion of single fingers consisting in the momentary increase of this state of flexion, which was immediately followed by a return to their former state; I saw this particularly often in the right index finger, but in others also. When I arrived, I was heated by quick walking, and a dinner which I had just taken, so that, when I felt for the pulse of the corpse, pulses beat everywhere, which in reality were to be found only in the points of my own fingers; this happened also to the assistant physician, who was present. Afterwards, as might have been expected, I could feel neither the pulse nor the heart beat. It appeared to me that the stimulants which excite pain were not well suited to follow up the traces of life in a cholera patient apparently dead; and I therefore contented myself with looking for traces of respiration by holding a lighted candle before the open mouth and nose; but the flame did not move. I now placed a glass

* We are afraid that a recovery in which the characters of youth and beauty were in a great measure lost, would scarcely be deemed a "perfect recovery" by the patient. But who can stand cupping for a twelvemonth?—Edit.

of water on the chest, on the right pectoralis major, and it was supported by the assistant physician. The glass moved indeed, not from respiration, but merely when the deltoid was convulsively agitated; and these were the most frequent of all the movements. Some particular muscles were contracted in preference, namely, the right deltoid (whose motions continued more than two hours and a half after death;) and, next to it, the supinator longus, and the radialis externus longus, the flexors of the fingers and toes. After some time I exposed a portion of the deltoid, near the pectoralis major, and saw that for the most part it had been only some few of the larger bundles of fibres which had moved. I then exposed the brachial artery, to see if there was any remnant of pulse; but, as might have been expected, there was none. I laid bare a larger portion of the crural artery, and a part of the sartorius muscle, but there was no pulse; and, as it had now grown late, there were no convulsions.

On the 2d of January, 1833, from one to half past one in the afternoon, I watched the convulsions in the body of a strong middle-aged man, who had died at half-past eleven. When I came, I found the corpse covered, and tolerably warm everywhere, but especially on the back. Several bundles of muscles were in convulsive movement on the left upper arm, particularly its lower portion; more especially the biceps, but also a part of the brachialis internus. This was also the case on the upper and anterior part of the left forearm; but here, from the slightness of the convulsions, it was not easy to distinguish what muscles were in motion. On the outer and upper part of the left thigh, the vastus externus moved, and on the anterior part the rectus. There were no motions of the fingers or toes; nor were any movements perceptible on the right side. I attempted, by rubbing the upper part of the right thigh, first with my hand, and afterwards with a brush, to produce convulsions in the rectus and vastus internus, and succeeded for a short time, but soon failed. The convulsions in the left thigh then ceased, and could not be restored by a piece of rag dipped in spirits of wine and set on fire, and those in the left arm soon disappeared likewise.—*Ueber den Leichenbefund bei der Orientalischen Cholera. Von Dr. P. Phœbus, Berlin,* 1833, p. 263 et seq.

### TREATMENT OF HYDROPHOBIA.

*Royal Academy of Sciences, September 23d.* A communication was read from M. Buisson, who states that the treatise on Hydrophobia sent in 1823 was written by him, and that the individual mentioned in it, as cured of hydrophobia, was himself. He regards his method as so certain, that he offers to inoculate himself with the disease. M. Buisson, who requests that his memoir may be allowed to compete for the Monthyon prize, gives the following history of his attack, and his cure.

He had been called to attend a woman who for three days had been suffering from what was supposed to be hydrophobia. She uttered piercing screams, complained of a sense of constriction in the throat, foamed at the mouth, and spit continually. The neighbours of the patient said that she had been bitten forty days before by a mad dog. She herself did not confess that she was labouring under hydrophobia, but maintained that the symptoms depended on the appearance of the catamenia. She was bled at her own earnest request, and died two hours afterwards; a termination which might have been foreseen before the operation. M. Buisson, whose hands were covered with blood, rubbed them with a towel which had been used to wipe the mouth of the patient. One of his fingers was affected at the time with ulceration depending on caries; yet he thought to protect himself from danger by subsequently washing with pure water. Nine days afterwards, being in a cabriolet, he suddenly felt a pain in his throat, and a still greater one in his eyes. His body seemed so light, that he felt as if he could leap to a prodigious height; and his scalp was so sensitive, that he thought he could count his hairs without seeing them. His mouth was filled with saliva, and the impression of the atmosphere, as well as the sight of shining objects, caused a very painful sensation. He also felt a desire of running and biting, not men, but animals and inanimate objects. Lastly, he drank with difficulty, and the sight of water oppressed him more than the pain in his throat. These symptoms returned every five minutes, and it seemed as if the pain began in the injured finger, and thence extended to the shoulder. The combination of these symptoms made him conclude that he was attacked with hydrophobia, and he resolved to put an end to his existence by suffocating himself in a vapour-bath. He increased the heat to 42° (= 126° Fahr.) and was then surprised and delighted to see all the symptoms cease. He went out of the room cured, dined copiously, and drank more than usual. Since then he has treated more than eighty bitten persons, in four of whom symptoms of hydrophobia had appeared. All have been cured, except a child, seven years old, who died in the bath.

The treatment which he prescribes for those who have been bitten consists in taking a certain number of Russian vapour-baths, and in sweating violently every night by means of blankets and featherbeds. The perspiration is augmented by drinking large quantities of hot decoction of sarsaparilla. To show the advantage of copious and continued perspiration in this disease, he relates the following anecdote. A relation of Grétry, the musician, was bitten by a mad dog, as well as a number of other persons, who all died by hydrophobia. This one, however, when he felt the first symptoms of the disease, began to dance night and day, saying that he wished to die merrily; and he recovered. M. Buisson quotes moreover, to the same purport, the old histories of *tarentism* cured by dancing. He also observes that the animals in whom hydrophobia is most frequently spontaneously developed, namely dogs, wolves, and foxes, do not perspire.—*Archives Générales, Septembre.*

CASE OF ERYSIPELAS OF THE FACE.

Wm. Hogbin, aged 18, was admitted into Luke's ward, under Mr. Stanley, Aug. 22. He states that he caught cold the preceding week, and that, two days before he applied to the hospital, he was attacked with painful tumefaction and redness of the face. His bowels have not been relieved for four or five days. He has great pain in his head, and can get no sleep at night; pulse quick, irritable, and somewhat hard. Is ordered twelve grains of jalap, with five grains of calomel, immediately; to be followed by a compound senna draught in three hours; and, after the operation of these medicines, a draught to be taken every six hours, composed of saline mixture, with one drachm of antimonial wine. The face to be fomented frequently with warm stoups.

23. Pulse has lost its hardness, though still irritable; bowels have been well opened; fancies himself better.

24. Has had no dejections to-day; great tumefaction of the eyelids; pain in the head unremitted.—Continue the saline medicines. The calomel and jalap powder to be repeated. An evaporating spirit lotion to be constantly applied to the head.

25. Appears better; the pain in the head has almost left him; pulse much less irritable.—Saline mixture, with sulphate of magnesia, every six hours.

27. Is ordered two grains of calomel at night, in consequence of his tongue being coated with a dirty white fur.

29. Is much better in every respect. Simple saline medicines alone to be administered.

September 1. Has been gradually improving until this morning, when he had a recurrence of the erysipelatous inflammation; the pain in the head returned; his pulse became quick and irritable, without much power.—His bowels to be opened with calomel and jalap powder immediately, and to continue the saline medicines.

2. Is somewhat better: pain in the head relieved since the operation of the purge.

4. Continues to improve. The bowels to be kept gently relaxed by saline mixture.

6. Has no pain in the head; the tumefaction and redness of the face have almost entirely disappeared; free dejections; pulse less irritable, but slightly intermittent.

12. The patient has been daily improving, and is now convalescent.

In this case, as in several others lately reported, the purgative and saline treatment alone has been found sufficient to remove this disorder; and depletion by venesection seems rather to retard than accelerate recovery, particularly in the instances where those labouring under this affection have been addicted to the use, or rather abuse, of fermented liquors and ardent spirits; and this fact is worthy of being noted, as frequently the bounding full pulse might seem, to one ignorant of this circumstance, to indicate the bold use of the lancet.—*Lancet.*

EFFECTS OF AN OBTURATOR MADE OF DIFFERENT METALS.

A lady, under the care of MM. Nauche and Moncourier, had a great fissure in the palate, through which her food used to pass into her nostrils. A dentist very ingeniously made an obturator which remedied the accident, but caused a remarkable sensation. A metallic taste and a slight numbness were experienced, analogous to those produced by galvanic arrangements. Suspecting that this might be occasioned by the dissimilar metals employed in the composition of the instrument, MM. Nauche and Moncourier put into their patient's mouth two disks of zinc and copper soldered together: the sensation now produced was recognized as similar to that caused by the obturator, differing only in intensity. The dentist was ordered to make another instrument, all of platina; the former being of platina jointed with gold. No sensation was experienced from the new obturator. From this we may gather the necessity of making those instruments of a single metal which are intended to be left in the system. When the metals are dissimilar, an electric current will be established, giving rise to decompositions of the fluids which they touch, and to feelings which may prove very troublesome.—*Med. Gaz. from Gazette des Hôpit.*

BAD RICE THE SUPPOSED CAUSE OF ASIATIC CHOLERA.

Dr. Tytler, a surgeon in the service of the East-India Company, has propounded his theory of the cause of Asiatic cholera to the London Medical Society, where it has afforded the subject for several nights' discussion. Dr. Tytler imagines that the Cholera Spasmodica, or (as he calls it) the Cholera Oryza, is produced by spoiled rice; and, by talking, writing, and thinking about this theory for some fifteen years in India, he has become so staunch a believer, that he has at last imported it from the East, where it might have some degree of plausibility, to England, where the theory has little but fancy to support it. As the investigation of one grievance very often tends to the discovery of another, we wish that this oryzean debate would incite some English Raspail to look at the state of what is called bread in London.

The following bit of dialogue at the Medical Society, which we take from the *Lancet*, is amusing enough:

"*Member.* The population of Jessore was two millions before the cholera occurred there, but what is it now?

"*Dr. Tytler.* Oh, it is still two millions.

"*Member.* Has the fatality caused by the cholera so speedily been—

"*Dr. Tytler.* God bless you! the destruction of people by the cholera in India is only like killing a bag of mosquitoes.

"*Member.* A most prolific climate. India is a wonderful country.

"*Dr. Tytler*. India is the world, sir.

"*Member*. Shall you return to it?

"*Dr. Tytler*. Yes, certainly.

"*Member*. Is not the climate too hot for you? I should think your substantial frame afforded a nice treat for the insects. [Dr. Tytler is a very robust, healthy-looking man.]

"*Dr. Tytler*. God bless you! I have been more stung in bed since I came to this nasty hole (London) than all the years I have been in India. See there: India is the country of humbugs, but this is the country for real bugs."

### A. L. WIGAN AND THE RINGWORM.

The lovers of novelty will be delighted to hear of the printed letter circulated by one A. L. Wigan, of Brighton. It comes to you by the twopenny post, and you are amazed to find, not a tailor's puff, nor a catalogue of a bankrupt's stock, selling off at awfully low prices, but a medical man's bran-new method of curing ringworm. Our brother of Brighton tells us that he sees at his dispensary nearly 150 patients weekly, and he has private practice besides: two elements of a problem far too knotty for us, and which we must submit, therefore, to our Cambridge friends. We suspect that its solution depends on the calculus of variations, as thus: "A man sees 150 patients weekly, &c.; how many cases of ringworm does he treat in a year?" We often see the practice of physic talked of in books as a harassing occupation, and no wonder; for just listen to A. L. Wigan: "During more than twenty years of my professional life, this disease was a constant source of disappointment and vexation to me." So that if a good long professional life consists of fifty years, here are two-fifths of it embittered by one tiny disease, which would hardly get two pages for its share in a system of physic: and then there remain a thousand maladies, which even A. L. Wigan himself cannot cure, to throw their bleak shade over the remaining three-fifths.

We must not conceal from our readers that there are difficulties in this letter; but difficulties that the author will no doubt clear up in the course of his correspondence with the world. Thus he tells us, that to ferret out a ringworm, we must look at it "*with a very powerful lens*; an examination with the naked eye is worth nothing at all;" but he does not tell us what we are to find with the lens. Moreover, "Ringworm is not only frequently mistaken for, but is often actually complicated with, porrigo, psoriatis guttata," &c. Now, on turning to Bateman, (who, though not a Wigan, is somebody,) we are sorely perplexed by finding that "The Porrigo *scutulata*, popularly termed the *Ringworm of the scalp*, appears in distinct and even distant patches." &c. (*Pract. Synopsis of Cutan. Diseases*, 6th edit. p. 169.) So that it may be mistaken for itself. Now for the treatment. "I believe that I am the first who adopted it—if not, it certainly was not suggested to me by any one," says A. L. Wigan. "The head then being shaven, and time given for any slight cut of the razor to be healed, I apply all over it (by means of a short soft shaving-brush) the strong pyroligneous acid prepared by Beaufoy, diluted with one third of its volume of water—keeping the head thoroughly wet with it for the space of two minutes. Some slight and very transient pain is given at the spots, *which are immediately rendered visible, though they could not previously be distinguished,*—they become instantly of a very bright red, while the healthy scalp is not at all affected, except in some rare cases, where the skin being exceedingly thin and delicate, it is advisable to use the acid in a more dilute state. The complete detection of every infected spot makes our further progress easy and satisfactory, as we know the extent of the disease. I now continue to *soak* the affected parts with fresh applications of the acid in its full strength for a quarter of an hour; and if this be done carefully and steadily, a second or third application (with intervals of three or four days,) will always complete the cure. Once is sometimes sufficient, but I do not like to trust to it." Now, when on the first of January, A. L. Wigan reads the following bit from Bateman, we are sure that he will cry out with old Donatus, *pereant qui nostra ante nos dixerint,*—the devil take those who said our good things before us. "Mr. P. Fernandez mentioned to me an instance of speedy recovery, which followed a single application of the strong sulphuric acid, which was instantly washed off. A new and healthy cuticle succeeded. The acetic acid, or aromatic vinegar, which acts as a more gentle, yet very effectual caustic, has proved an effectual remedy in a few instances."—(*Bateman's Synopsis*, 6th edition, p. 173. Note.) We now take leave of the author. We do not object to his manner of treating ringworm, but we object most strongly to his manner of communicating his treatment. Would none of the Brighton doctors listen to him, or come and see his cures? It would have been a milder step to ask them to do so. "Mr. Wigan presents his compliments to Dr. Quick, and begs the favour of his company to a curing of ringworm, with acetic acid." "Dr. Quick is very sorry that he cannot wait upon Mr. Wigan, as he has invited a medical party to see him cure two aldermen of dyspepsia crapulosa, with the sulphate of zinc."

Or, why could not the author send his discoveries to the "Medical Gazette," or the "Lancet?" He should reflect, that if his unhappy example should be generally followed, everybody communicating his practice to everybody by the post, we poor doctors must certainly make our appearance in the Gazette, not the Medical, but the other one.

### BINELLI'S SOLUTION AND KREOSOT.

It is difficult to decide on the merits of new remedies, for experiments on men can be tried only in certain cases, and when made on animals, they give merely approximate results. In the instance of Binelli's solution, there was an additional difficulty, namely, that the composition was unknown. Excepting in Italy, where the commissions appointed by the governments at Turin and Naples declared themselves in favour of this secret remedy, it was first tried in Berlin, in the year 1831. As the experiments (which, however, were not very numerous,) terminated favourably without exception, they were published, with the promise that everything should in due time be communicated which might be learned from further investigation in the institution. We now discharge this obligation, as far as we can. Through the kindness of Count Lottum, the Prussian ambassador at Naples, we received a large quantity of this solution from the fountain-head. The bottles differed from one another in smell and appearance so much, as to give rise to rational doubts as to the sameness of the preparation. The intensity of these doubts was increased both by recent intelligence from Naples, stating that Captain Pironti, the present possessor of the secret, was not conversant with chemistry, and by the fact, that the experiments undertaken here in 1832 were not satisfactory. It resulted from them that Binelli's solution was an uncertain styptic both in animals and men. In a few cases it appeared efficacious, but in many others was of so little use, that we were obliged to resort to other aids; and in an amputation of the breast, and a removal of the humerus, we were obliged to tie the vessels. Our confidence in the new remedy was of course much lessened by these occurrences, as well as by the counter-experiments performed at the institution, in 1832; they showed that in rabbits, sheep, and horses, when the large arteries, and even the carotids were divided, the bleeding could sometimes, though rarely, be arrested by plugs of lint, or by cold water; and sometimes without any application. It is a uniform and perfectly certain operation alone which can give value to the preparation in question. It remains for time to decide whether this effect can be produced by a scientific employment of the peculiar ingredient contained in this arcanum;—an ingredient which has hitherto been too little investigated. It would be too hasty to drop the thing altogether, on account of the less satisfactory nature of our last experiments. Many distinguished Italian physicians, among whom I will name only Cotugno, Antonucci, Santoro, Boccanera, Ronchi, Vulpes, and Rispoli, have seen the internal and external use of Binelli's solution, of singular advantage in wounds where a ligature could not be applied, as well as in cases of hemoptysis and metrorrhagia. Some Berlin practitioners have similarly employed, and with advantage, the arcanum in imitation of Binelli's solution, prepared by the apothecaries Hummel, Jaenicke, Daneel, Staegemann, and Leide.

The late discovery of Kreosot, to which professor Schweigger Seidel had the kindness to direct my attention, will perhaps once be the means of the contradictory results of the experiments. This savant supposes Kreosot to be the peculiar substance which Berzelius discovered in the solution which we sent him; it possesses the power of coagulating the albumen in the blood, and it is to Kreosot that smoke and pyroligneous acid owe their conservative powers.— *Gräfe, in his Journal.* Band xx. Heft 2.

TREATMENT OF CHOLERA BY CALOMEL ALONE.

We have received a letter from Dr. Peacock, of Darlington, who informs us that, in cases of Indian cholera, he gives a grain of calomel every ten minutes, with a glass of cold water; that he trusts to this alone, having found the saline mixture a useless addition, and that he is uniformly successful. Our correspondent concludes by saying, " I am so well convinced of the utility of this plan of treating the Indian cholera, that, if any two respectable practitioners will make a full and patient trial of it, either in England or America, I will be bound to forfeit a sum of money to any public charity in every instance in which it fails."

This will remind our readers of Dr. Ayre's system, who gives a grain of calomel every five minutes, but combines it with a drop of laudanum. Dr. Peacock's proposed bet is a very rash one; we should be glad to hear if any of the sporting doctors of the North have taken it.

CASES OF CLIMACTERIC DECAY.

A gentleman had been for some years attended by the writer. At the age of eighty-one years, during a severe winter, he suffered much from bronchitis, accompanied with great sinking of the vital energies. His habits were social, and he lived highly. He recovered, however, by means of warm diaphoretics and tonic cordial aperients, with a due regard to his accustomed indulgences, and to the precept of Hoffmann, "*ne subito muta assueta, quia assuetudo est altera natura.*" The following year he had a similar attack at his seat in the country. A nearly opposite treatment to that which was adopted by the writer in his previous illness was directed by his medical attendants on this occasion, and in a few days he expired *when seated* on the nightstool, [see Hoffmann's treatise "*De Situ erecto in Morbis periculosis valde noxio,*"] about half an hour after the physician had left him, and given a favourable opinion of the result to his friends.

General —— had served nearly all his life in the East Indies, and was upwards of eighty, but of a robust constitution. His ailments, when he was seen by me, could not be referred to any particular organ, and were attributed at the time to senile decay: the liver performed its functions. Nothing beyond the regulation and promotion of the digestive and excreting functions was attempted; and he was allowed a light and nutritious diet, with change of air, the use of the Bath water, &c. Under this plan he improved greatly, and was able to travel with ease from one part of the country to the other, and, when in town, to dine daily at the Oriental Club. The last occasion but one on which I saw him, he came to my house, to inform me that his relatives were not satisfied with the progress he had made, and had repeatedly urged him to change his physician. I accordingly retired; but a few days afterwards was requested to see him. He was then sinking fast, evidently from the effects of a lowering treatment and of profuse evacuations upon a decayed frame. Speedy dissolution could not be averted; I therefore declined all interference. He died not many hours afterwards.—*Dr. Copland's Dictionary of Practical Medicine.*

### POST-MORTEM EXAMINATION OF A PAINTER.

Mr. Byam and myself recently examined the body of a painter, who died at the age of seventy-eight. He had been a very strong man, and in constant employment all his life, up to a few days before his death. He died of hæmatemesis, from disease of a branch of the coronary artery of the stomach. The substance of the heart was soft and flabby. The small and large intestines were sound; the liver was studded with collections of a pultaceous semi-fluid matter, of a greyish white colour, contained in very thin cysts, from the size of a hazel-nut to a walnut, the portions of liver surrounding them being softened, and of a dark red colour. The top of the anterior mediastinum, and space behind the top of the sternum, contained an immense mass, nearly the size of the closed hand, of enlarged glands, of a cheesy consistence and appearance: and a similar change of the absorbent glands existed behind the arch of the aorta, the superior cava, &c., extending in the form of a long cushion down the vertebræ into the abdomen. The small arch of the stomach, the pylorus, and commencement of the duodenum, were remarkably thickened, from the deposition of adventitious matter, the thickened mass nearly approaching the characters of scirrhus. The coats of the arteries of the stomach were diseased, and contained atheromatous matter.—*Ibid.*

### THE CRETINS OF SALZBURG.

The following account of the "*Fexes*," or cretins of Salzburg, is abridged from that given by Professor Knolz:

The whole body is stunted, its height not exceeding four feet. There is a total want of due proportion between its different parts; the height of the head, with reference to the rest of the body, being one fourth or one fifth, instead of one eighth, the natural proportion. The neck is strong, and bent downwards. The mammæ are very voluminous and pendent; the upper limbs reach below the knees; the arm is shorter than the forearm; the chest narrow; the abdomen hemispherical, and of a length not exceeding the height of the head; the penis and scrotum come down to the knees; the thighs are, with the haunches, of a greater width than the shoulders, and are shorter than the legs, the calves being almost wanting; the foot is small, and the toes partly distorted; the lower extremities are shorter than the upper half of the body. In the head, the masticating organs, the lower jaw, and the nose, preponderate considerably over the organs of sense and intelligence. The skull is depressed, and forms a lengthened and angular ellipsis; the receding forehead presents, internally, large frontal sinuses, to which the brain has yielded a part of its place; the top of the head is not vaulted, but flattened; the occiput projects but slightly, and runs almost even with the nape of the neck, as in ruminating animals. The face is neither oval nor round, but spread out in width; the parts of which it is composed being wide and short, and the maxillary bones projecting greatly. The forehead is narrow, flattened, and low; the eyes are unusually far apart, diverge slightly, and are small, and seated deep in the orbit; the pupil is contracted, and not very sensitive to light; their external angles are situated higher than the internal; the eyelids, unless when dropsically swollen, are flaccid and pendent; the look is a fixed stare without expression, and turns with indifference from all that is not eatable. The root of the nose is widened and depressed, the bones of the nose square; the zygomatic bones are wide, and extremely projecting; the external ear is large, stands out from the head, and hearing is very defective. The elongated form of the lower jaw of the cretins, and their thick and padded lips, make them resemble ruminating creatures more nearly than man. The tongue is thick, and rather cylindrical than flat; the saliva is continually running from the angles of the mouth. Enlargement of the thyroid gland is recognized as one of the signs of cretinism; but its size is no sure guide to the extent of the existing infirmity. The throat presents also other obstructed glands. The thorax is generally narrow and flat; the abdomen is usually distended with gases, and largely developed towards the chest; the flesh of the extremities is flabby; the knee of an irregular shape, and usually bent; the fingers are very long and lank, and the nails very small. The upper part of the vertebral column being directed more or less forward, and the lower part, with the basin, being pushed backward, the sacrum assumes a more horizontal, and the other pelvic bones a more vertical position, than in the healthy formation. Besides the masticating and digestive organs, those of generation are also strongly developed, especially in the male. (*Medecin. Jahrbücher des k. k. Œsterr. Staates*, b. i. st. 1, 1829, p. 86.)—*Ibid.*

### STEEL BUSKS.

It is extremely possible that whatever *conducts the electricity* of the body from it, will occasion direct debility. With this view I have long been in the habit of causing females who used steel supports to their stays, to lay them altogether aside. The experiments on Casper Hauser confirm this supposition.—*Ibid.*

### CASE OF APOPLEXY.

Travelling in the summer, in one of the short stages, I sat opposite an aged and corpulent man, who, very soon after our leaving town, suddenly lost his consciousness and power of motion. His countenance became first pale, then bloated and inexpressive, his breathing slow and slightly stertorous, all his muscles completely relaxed, and he fell, in a few seconds, upon those sitting around him. We were only a few doors from a chemist's shop; the coach was stopped, and he was carried thither. He was now profoundly apoplectic; a copious perspiration flowed from his face and forehead, the veins of which were distended, and all his senses were completely abolished. There was no sign of hemiplegia, but there was general and complete loss of motion and sensation. His neckcloth having been removed, the pulsation of the carotids was found to be slow, and of natural strength and fulness. Whilst he was held in a sitting posture in a chair, cold water was poured gently over his head from a sponge, and his head frequently sponged with it; volatile salts also were held for a short time, and, at intervals, to his nostrils. The power of deglutition was at this time abolished, so that it was impossible to administer a draught, chiefly consisting of a small quantity of spiritus ammoniæ aromaticus and camphor mixture, which was prescribed. In a very few minutes his consciousness returned, he took the draught, and in a short time afterwards he walked to a coach, in which I accompanied him home. He now complained only of very slight confusion of ideas, with scarcely any headach, but his carotids beat more firmly. One full bloodletting, and an active purgative, were now directed. The next day he was perfectly well, and has continued so. What would have been the result if he had been largely blooded previously to the reaction?—*Ibid.*

### MEDICATED BATHS.

The idea that the skin is entirely incapable of absorbing fluids in which it may be immersed, has led to the neglect of medicated baths. But it should be recollected that, independently of any power of absorption this structure may possess, and which I believe it possesses under some circumstances, and in respect of various agents, it is a living, an active, a finely sensible, and, as to the nature and extent of its functions, an important organ ; and that it is very susceptible of impressions by which not only its own functions are modified or altogether changed, but the actions of other organs are variously affected in consequence of the nervous and vascular connexions and functional relations, which bind the different parts of the economy into one indivisible whole. Entertaining such views, I believe that cold, tepid, warm, or medicated baths; that lotions or washes, or stimulating liniments and frictions applied to the surface, the former in slighter cases, the latter in the more urgent; are not infrequently beneficial in diseases of debility, when judiciously employed, and with due reference to antecedent or existing visceral disorder. Sea or salt water bathing; shower baths; camphor and chalybeate baths; warm, tepid, or cold baths, either general or local, of iodine, or of iodine and sub-carbonate of potassa; baths of decoctions of willow or oak bark, sometimes with the addition of an alkaline sub-carbonate; washes with camphor water, rose water, and vinegar, applied to the trunk; or sponging the surface daily with a mixture of these, at a temperature of about 60°; or with a small proportion of the nitric and muriatic acids in water at a temperature of 70° to 80°; are respectively of much service, when suitably prescribed.—*Ibid.*

### TREATMENT OF ASCITES CONSEQUENT ON AMENORRHŒA.

I was consulted, some years ago, respecting a case of ascites consequent upon profuse and frequent menstruation. This discharge had been suppressed by exposure to cold; and, soon afterwards, symptoms of inflammation of the serous covering of the liver, with effusion, were observed. These were combated by local depletions, which were repeated; by external irritants, by mercurials, and, subsequently, by cream of tartar with borax and diuretics, and other means in various forms of combination; but without any permanent benefit. I directed at last a weak solution of the hydriodate of potash with iodine; and caused it to be persisted in for seven or eight weeks, when good effects began to appear. This medicine was continued for five or six months; at the end of which time the catamenia had become regular, and the effusion had entirely disappeared. I was more recently consulted as to a similar case, in the care of Mr. Grabham, of Rochford; which had, likewise, been preceded by profuse catamenia, suppression of this discharge followed by pulmonary disease, and extension of tenderness and fulness from the thorax, over the region of the liver and abdomen; with effusion of fluid into the abdominal cavity. The pulmonary affection and the more acute symptoms subsided under the very judicious practice of this gentleman; but the means successively adopted in consultation failed of removing the dropsical collection, and of arresting the progressive emaciation. There was also, in this case, scrofulous disease of one or two of the metacarpal bones of the left hand. This was left to itself, in hopes that the discharge from it would have had a salutary effect on the principal seat of disease. In the summer, 1832, this young lady came to London, where various remedies were prescribed, without relief. I then put her upon a course of iodine; and, directing her to persist in its use, advised her return to the country. I have since understood that, during the use of this medicine, the effusion disappeared, and the catamenia returned; that she recovered her looks, and is now married.—*Ibid.*

### TRANSFERENCE OF VITAL POWER.

A not uncommon cause of depressed vital power is the young sleeping with the aged. This fact, however explained, has been long remarked, and is well known to every unprejudiced observer. But it has been most unaccountably overlooked in medicine. I have, on several occasions, met with the counterpart of the following case: I was, a few years since, consulted about a pale, sickly, and thin boy, of about five or six years of age. He appeared to have no specific ailment; but there was a slow and remarkable decline of flesh and strength, and of the energy of all the functions, what his mother very aptly termed a gradual blight. After enquiry into the history of the case, it came out that he had been a very robust and plethoric child up to his third year, when his grandmother, a very aged person, took him to sleep with her; that he soon afterwards lost his good looks; and that he had continued to decline progressively ever since, notwithstanding medical treatment. I directed him to sleep apart from his aged parent, and prescribed gentle tonics, change of air, &c. The recovery was rapid. But it is not in children only that debility is induced by this mode of abstracting vital power. Young females married to very old men suffer in a similar manner, although seldom to so great an extent; and instances have come to my knowledge, where they have suspected the cause of their debilitated state. These facts are often well known to the aged themselves, who consider the indulgence favorable to longevity, and thereby often illustrate the selfishness which, in some persons, increases with their years.—*Ibid.*

### CONVULSIONS ACCOMPANYING THE CUTTING OF SECOND TEETH.

Although convulsions, or epilepsy, be not so common during the progress of second dentition as they are in the earlier development, yet many cases occur in which their presence may be attributed to pressure, opposing the progress of growth, and thus irritating certain nerves.

A boy, twelve years of age, was cutting the second or posterior permanent molares of the upper jaw, before those of the lower, and the process was accompanied by twitchings of various parts of the body. At last he became affected with chorea. Being a very nervous lad, if any notice were taken of him, he would quite involuntarily make the most extraordinary faces, and contort his body into various attitudes, that appeared to be most difficult and painful. This chorea continued for three months, during which time a variety of medicines were swallowed. At last he fell into an epileptic fit, struggling much, foaming at the mouth, and grinding the teeth. I thrust my fore-finger along the inside of his cheek, and found a hard cartilaginous space on each side, behind his first molar teeth. I succeeded in gashing these parts: he uttered a scream, and fell out of his fit, becoming quite sensible; nor had he a recurrence of his chorea.

A young gentleman, twelve years of age, with dark hair and eyes, greasy countenance, lymphatically stout, was removed from school, and sent to some relations in the country on account of being attacked by chorea. His bowels were costive; he had offensive breath; he started and moaned in his sleep. Something unpleasant to his feelings occurred in conversation, and he fell into an epileptic fit. On recovering, he was placed in bed, where he slept soundly for two hours, and on awaking was perfectly unconscious of having had a fit. During the next fourteen months, this boy had many recurrences of his fits, and though much medicine was swallowed, nobody thought of the probability of his cutting teeth. In shewing me the young man at eighteen, his father said he had had no fits for four years: he supposed he had "outgrown" them, a truth spoken without reference to my hypotheses.

A lady, with dark hair and eyes, tall and handsome, of a highly nervous temperament, married at the age of nineteen; and was pregnant of her first child about eight months, when she was seized with an epileptic fit. She had for several months been affected with twitchings in her cheeks, and "live blood" was often very troublesome in her left eye. The epilepsy recurred twice before her labour, which was a very favourable one; and she had a very good getting-up. She nursed her child, which throve very well. At the end of seven months from her delivery, twitchings came on again, and she had at last another epileptic fit. They came on now twice, sometimes three times, in the week. The best physicians, some of the best surgeons, were consulted. The digestive organs were attended to, and she took various medicines. The fits came on at longer intervals; then, sometimes, two appeared in a day. I saw her first when she was twenty-two years of age. I was expressly told that she was placed under my care, with no hope of her recovery, but merely that I might look to the general regulation of her health. My first office was that of consulting-dentist, for I gave her an opinion upon her teeth, which was to the effect of condemning her to lose seven decayed molares. One of these was the only wise tooth she had cut. Three other hard cartilaginous obstacles to the progress of her teeth were removed, and this lady has had several years of freedom from epilepsy.

A young woman, nineteen years of age, of light hair and fair complexion, of fine tall figure, rather fat, was, in the year 1818, apprentice in a straw-bonnet shop, in Hayes-court. Her occupation was sedentary, and she had not been in the habit of paying attention to the state of her bowels: they were suffered to be very costive. For several months she had perspired profusely at night, and her breath had been observed to be very offensive; she started in her sleep, and had repeatedly awakened her bedfellow by kicking her on these occasions. She moaned and talked in her sleep. Dr. Nuttall, who was my colleague at the Westminster General Dispensary, was suddenly called to her, on account of her having fallen into a fit. He caused her to be profusely bled, and she recovered so far as to be able to see her physician at the dispensary. Three weeks afterwards, the doctor being from home, I was obliged to see this patient, in a fit similar to the first she had had. I learned that she had been very odd and nervous in her manner, and had often suddenly screamed out from cramps seizing her toes and the calves of her legs, which were succeeded by her thumb being drawn in towards the palm of her hand, and her fingers being clenched upon it. I found her in a state of tetanus. The convulsions was over. I thrust my fore-finger into her mouth, where I found the wise teeth of the upper jaw through. In the lower jaw the teeth could not get through, for there were hard cartilaginous substances in their way; through these I scored freely, and the young woman was relieved instantly.

I was called last year to Upper Marylebone-street, in the night, to see the daughter of a very honest and independent tradesman. She had been attended for the fit in which I saw her by two medical friends. The parent's anxiety made him apply to me. The patient was a fine young woman, of nineteen, with dark hair and eyes. She was stiffly curved in tetanus. I asked her sister if she knew whether any wise teeth had lately been cut. Upon her answering in the affirmative, I took a gum lancet, and relieved my patient instantly, by cutting through the obstacle to the progress of her wise tooth, in the right side of the lower jaw.—*Dr. Ashburner on Dentition, in Med. Gazette.*

### A CASE *of* ANASTOMOSING ANEURISM *of the* EXTERNAL MAXILLARY ARTERY, *treated successfully by Tying the Common Carotid Artery.*

By David L. Rogers, m.d. Lecturer on Surgery. Communicated by S. R. Kirby, m. d.

This case occurred in a child, aged eight months, at the time of the operation. At its birth a small pulsating tumour was observed in the centre of the right cheek, which continued to enlarge, until it embraced nearly the whole of it.

It was bounded above by the prominent part of the malar bone; below by two thirds of the inferior edge of the inferior maxillary bone; posteriorly by the superior part of the inner edge of the sterno-cleido-mastoid muscle, on a range from above downwards, with the lobe of the ear; anteriorly, by a line drawn from the inferior part of the nostril, and terminating about one inch from the symphysis of the chin. The tumour pointed in two places, just above the ear, and at the angle of the jaw. It was irregularly convex, having its greatest convexity at its posterior part, and gradually diminishing from behind forwards; its colour purplish, with several red spots on its surface. The child seemed otherwise in good health. The operation was performed in the presence of Drs. Mott, Baxter, and Kirby, on Thursday, December 12th, 1832. An incision was made through the skin and the platysma myoid muscle, of about one and a half inches in length, in the direction of the inner edge of the sterno-mastoid muscle, but nearer to the trachea than to this muscle, which kept the external jugular vein at a greater distance; at the first incision a small artery was divided, which was secured by a ligature; the adipose tissue was cleared away, and very soon the sterno-thyroid muscle was partially exposed, at the outer edge of which the sheath of the vessels was seen; this was punctured, and the artery secured with one ligature. No other vessels were tied, and the quantity of blood lost did not exceed a wine-glassfull. In a short time a diminution of the tumour was perceptible. A stitch was made in the middle of the incision, and adhesive straps applied. The little sufferer was not much exhausted; it was placed in its mother's arms, and immediately began to nurse, with occasional restlessness. A gradual diminution of the tumour continued until it had entirely disappeared, and the child wholly recovered, and is now in good health.—*The American Journal of the Medical Sciences, Nov.* 1833.

---

